# Prediction of Adverse Drug Reaction Linked to Protein Targets Using Network-Based Information and Machine Learning

**DOI:** 10.3389/fbinf.2022.906644

**Published:** 2022-07-14

**Authors:** Cristiano Galletti, Joaquim Aguirre-Plans, Baldo Oliva, Narcis Fernandez-Fuentes

**Affiliations:** ^1^ Department of Biosciences, U Science Tech, Universitat de Vic-Universitat Central de Catalunya, Barcelona, Spain; ^2^ Department of Physics, Network Science Institute, Northeastern University, Boston, MA, United States; ^3^ Department of Experimental and Health Sciences, Structural Bioinformatics Group, Research Programme on Biomedical Informatics, Universitat Pompeu Fabra, Barcelona, Spain

**Keywords:** network biology, drug adverse reaction, drug target, machine learning, protein-adverse reaction association

## Abstract

Drug discovery attrition rates, particularly at advanced clinical trial stages, are high because of unexpected adverse drug reactions (ADR) elicited by novel drug candidates. Predicting undesirable ADRs produced by the modulation of certain protein targets would contribute to developing safer drugs, thereby reducing economic losses associated with high attrition rates. As opposed to the more traditional drug-centric approach, we propose a target-centric approach to predict associations between protein targets and ADRs. The implementation of the predictor is based on a machine learning classifier that integrates a set of eight independent network-based features. These include a network diffusion-based score, identification of protein modules based on network clustering algorithms, functional similarity among proteins, network distance to proteins that are part of safety panels used in preclinical drug development, set of network descriptors in the form of degree and betweenness centrality measurements, and conservation. This diverse set of descriptors were used to generate predictors based on different machine learning classifiers ranging from specific models for individual ADR to higher levels of abstraction as per MEDDRA hierarchy such as *system organ class.* The results obtained from the different machine-learning classifiers, namely, support vector machine, random forest, and neural network were further analyzed as a meta-predictor exploiting three different voting systems, namely, *jury vote*, *consensus vote*, and *red flag*, obtaining different models for each of the ADRs in analysis. The level of accuracy of the predictors justifies the identification of problematic protein targets both at the level of individual ADR as well as a set of related ADRs grouped in common system organ classes. As an example, the prediction of ventricular tachycardia achieved an accuracy and precision of 0.83 and 0.90, respectively, and a Matthew correlation coefficient of 0.70. We believe that this approach is a good complement to the existing methodologies devised to foresee potential liabilities in preclinical drug discovery. The method is available through the DocTOR utility at GitHub (https://github.com/cristian931/DocTOR).

## 1 Introduction

Protein–protein interactions are central to all aspects of cell biology, including processes linked to diseases. The phenomenal technological development in recent years allowed the comprehensive charting of the protein–protein interactions that take place in human cells, the interactome [(Gavin et al., 2011; Xing et al., 2016; Xiang et al., 2021)]. Indeed, high-quality and high-coverage protein interaction maps are now available for a number of model organisms, including humans (Kotlyar et al., 2022). Such resources present a number of opportunities to the pharmaceutical industry, which can exploit this information to, for instance, identify plausible therapeutic targets from which to develop or repurpose drugs [as in the most recent case of COVID-19 drug race (Sahoo et al., 2021; Gysi et al., 2021)]. At the same time, these recent advances have also led to increased efforts to fill the gap of toxicology or safety information for drug's targets. This problem has always crippled the development of novel drugs, increasing the attrition of the latter entering clinical trials due to the severity of adverse drug reactions (ADRs) associated with unforeseen toxicity, directly increasing the cost of research (Seyhan, 2019).

Currently, several drug-centered approaches exist that can be used to reduce the risk of ADRs associated with novel drugs (Basile et al., 2019), such as the use of animal models (Bailey et al., 2014) and *in vitro* toxicology research (Madorran et al., 2020). However, these approaches involve high maintenance costs and ethical limitations and are not always transferable to human biology (Singh and Seed, 2021). Many *in silico* approaches have also proved to be useful in estimating the toxicity of drug candidates, exploiting features such as composition, structure, and binding affinity [(Lo et al., 2018), (Bender et al., 2007)]. These methods include various examples of machine learning (ML) and deep learning (Dara et al., 2022). Contributing to these efforts, we recently described the T-ARDIS database (Galletti et al., 2021). T-ARDIS is a curated collection of relationships between proteins and ADRs. The associations are statistically assessed and derive from existing resources of drug-target and drug-ADR association (Galletti et al., 2021). Since T-ARDIS provides a direct link between proteins and ADRs, the question arose of whether this information can be exploited to predict potential ADR linked to proteins. Therefore, the major driver of this project was to develop a target-centric approach to predict whether the targeting of a given protein target is likely to result in ADR using the curated information to train machine-learning classifiers.

To that end, different machine-learning classifiers were assessed including support vector machine (SVM), random forest (RF), and neural networks (NN). Highly significant associations between proteins and ADRs were extracted from T-ARDIS and characterized using 8 different features. These include the following: 1) the network diffusion-based score from GUILDify (Aguirre-Plans et al., 2019); 2) several network-based clustering algorithms [(Cao et al., 2014), (Blondel et al., 2008)]; 3) a functional similarity index; 4) network distance to proteins that are part of safety panels used in preclinical drug development; and 5) network descriptors in the form of degree and betweenness centrality measurements and conservation. All of the measurements use network-based information in some way and hence incorporate aspects that are intrinsic not only to the protein but also to the network. As a result, the proteins are framed within the interactome, and the potential impact of changes on neighboring proteins is assessed.

According to the MEDDRA nomenclature (Chang et al., 2017), specific models were built for each individual ADR, as well as clusters of ADRs within the same system organ class (SOC), allowing the analysis to be extended to a more general anatomical or physiological system. Besides the datasets derived from T-ARDIS to train and test the models, we also benchmarked our prediction in independent datasets including manually curated dataset compiled from literature [(Huang et al., 2018), (Mizutani et al., 2012), (Smit et al., 2021), (Kuhn et al., 2013)—Supplementary Table S2], including a dataset submitted to the critical assessment of massive data analysis competition (Aguirre-Plans et al., 2021). Finally, as three different machine-learning predictions were developed, we also explored the accuracy of a meta-predictor that combines the predictions of each individual classifier. Three different meta-predictors were assessed based on the way the predictions were combined: 1) *jury vote,* 2) *consensus*, and 3) *red flag*. While *jury vote* and *consensus* scoring function are similar and seek to promote associations with high scores, *red flag* takes into account the divergent opinion.

The proposed method achieves a high level of reliability. For example, taking into account the undesirable effect of atrial fibrillation, the resulting model scored high in accuracy (0.88), precision (0.87), recall (0.85), and Matthew correlation coefficient (MCC) (0.77) for both the SVM and RF approaches. The neural network gives slightly lower results with 0.66 accuracy, 0.71 precision, and an MCC of 0.34. The obtained meta-predictors achieved similar results in jury voting and consensus methods with accuracy 0.89, precision 0.89, recall 0.88, and MCC 0.78. To be noted, the reliability of the model is closely related to the biological complexity and tissue specificity of various ADRs. The dataset employed in this study as well as the models, meta-predictors, and accessory scripts are available at https://github.com/cristian931/DocTOR. Upon installing the application, users will be able to upload a list of proteins in order to assess their relationship with the studied ADR.

## 2 Materials and Methods

### 2.1 Datasets

#### 2.1.1 Training Set

The set used to train and cross-validate the models was derived from T-ARDIS (Galletti et al., 2021). T-ARDIS is a database that compiles statistically significant relationships between proteins and ADRs. As described in original publication, T-ARDIS undergoes a series of filtering and quality control steps to ensure a reliable and significant relationship between the ADR and the protein targets. Depending on the source of ADRs associations used to derive target ADRs relationships, two groups were defined: relationships derived from self-reporting databases FAERS (Kumar, 2018) and MEDEFFECT (Re3data.Org, 2014); and relationships derived from curated databases SIDER (Kuhn et al., 2015) and OFFSIDES (Tatonetti et al., 2012). Both groups have been used to obtain the training set used in this work. For the self-reporting dataset, T-ARDIS currently contains about 17k paired protein–ADR interactions, including 3k adverse reactions and 300 Uniprot ids. The smaller curated dataset contains approximately 3,000 pairwise associations for 537 adverse events and 200 proteins. From the initial list of approximately 500 ADRs, only the 84 that were best characterized in terms of number of proteins associated and that covered the entire range of SOC classes, as defined by MEDDRA (Chang et al., 2017), were considered, i.e., included at least 5 numbers of ADR per SOC.

#### 2.1.2 Independent Test Datasets

For external validation, we employed five different independent datasets sourced from literature containing protein–ADR relationships from Kuhn et al. (2013)—Supplementary Table S2, Smit et al. (2021), Mizutani et al. (2012) the ADReCs-Target database (Huang et al., 2018), and the DisGeNet Drug-induced Liver Injury dataset (Piñero et al., 2019). In particular, the latter contains a specific subset of liver injuries caused by drugs composed by 12 different MEDDRA-defined events ranging from “Acute hepatic failure” to “Non-Alcoholic Steatohepatitis.”

More than 600 distinct adverse events and 428 proteins were retrieved, resulting in a total of 15 k interactions. Then, the 84 selected ADR were extracted, resulting in 188 associated proteins. The independent and the training dataset are totally independent in the sense that they do not share proteins between them on each particular ADR.

### 2.2 Protein Network

The protein network, or interactome, used in this study, was integrated using BIANA (Garcia-Garcia et al., 2010) and GUILDifyv2 (Aguirre-Plans et al., 2019). The original BIANA network includes interactomic information from IntAct (Kerrien et al., 2006), DIP (Wong et al., 2015), HPRD (Keshava Prasad et al., 2008), BioGrid (Stark et al., 2006), MPACT (Güldener et al., 2006), and MINT (Ceol et al., 2009) databases. The most recent version composed of 13,090 proteins (or nodes) and 320,337 interactions (or edges) has been used in this work.

### 2.3 Features

#### 2.3.1 GUILDify Score

GUILDify is a web server of network diffusion-based algorithms used for a wide range of network medicine applications (Aguirre-Plans et al., 2019). The message-passing algorithms of GUILDify (Guney and Oliva, 2012) transmit a signal from a group of proteins associated with a phenotype or drug (known as seeds) to the rest of the network nodes and score them depending on how fast the message reaches them, taking into account several network properties. Originally, GUILDify had been developed to prioritize gene–disease relationships and identify disease modules (Aguirre-Plans et al., 2019), but it was recently used to identify disease co-morbidities and drug repurposing options (Aguirre-Plans et al., 2019; Artigas et al., 2020). In this study, GUILDify was used as a feature to predict protein–ADR associations. Upon expansion, a GUILD score was assigned to each protein in the interactome based on the ADR's linked protein used as the seed. The higher the score, the more likely that an association exists between the protein and the set of seeds used to expand.

#### 2.3.2 Degree and Betweenness Centrality

Degree and betweenness centrality are two network analysis measures. Degree centrality is the number of edges connected to a node, while betweenness centrality is the number of times a node acts as a bridge along the shortest path between two other nodes. Both measures define how relevant a given node is inside a network and, in terms of the interactome, how much a protein tends to be part of a cascade of signals and participate in the same biological process. Degree and betweenness centrality values were computed using NetworkX (Ceol et al., 2009).

#### 2.3.3 Clustering-Based Algorithms

Another interpretation of the “guilt-by-association” principle is the definition of “disease module,” i.e., a neighborhood of a molecular network whose components are jointly associated with one or several diseases or risk factors (Choobdar et al., 2019). As shown, disease modules can be used to identify protein/genes associated with given diseases (Goh and Choi, 2012). In the context of ADRs, the assumption is that proteins linked to the same ADRs would cluster in local regions of the interactome, forming ADR modules (Guney, 2017).

To identify these modules, two different clustering algorithms were used. First, the K1 clustering algorithm is based on the so-called diffusion state distance (DSD) metric (Cao et al., 2014). The DSD metric is used to define a pairwise distance matrix between all nodes, on which a spectral clustering algorithm is applied. In parallel, dense bipartite subgraphs are identified using standard graph techniques. Finally, results are merged into a single set of non-overlapping 858 clusters. The second clustering method is based on the work by Lefebvre and col ((Blondel et al., 2008)), which is based on modularity optimization, assigning, and removing recursively the nodes to the modules found, each time evaluating the loss or gain of modularity. We applied this method to the interactome, retrieving 46 modules. Together with clustering approaches mentioned above, we compute for each node the “clustering coefficient” using the NetworkX utility (Ceol et al., 2009).

#### 2.3.4 Function Conservation Index

A new feature included in the newer version of GUILDify is the identification of enriched Gene Ontology (GO) functions among top ranking proteins using Fisher’s exact test (Aguirre-Plans et al., 2019). The function conservation index, which takes advantage of this resource, considers the functional similarity between a protein and GUILDify’s enriched GO terms. In a nutshell, this value is the result of a Hamming distance between two binary vectors that represent the presence or absence of a specific GO term. The shorter the distance, the higher the similarity between the given protein and the enriched functions identified from a set of protein–ADRs. The scale represents the ratio where a 1 would indicate full overlap of functions.

#### 2.3.5 Shortest Path to Very Important Targets

Targets and pathways that are now well established as contributors to clinical ADRs are included in safety panels, which constitute the minimal lists of targets that qualify for early hazard detection, off-target risk assessment, and mitigation. (Bowes et al., 2012a). Here, we considered the Safety Screen Tier 1 panel of EuroFins Discovery based on the work by Whitebread and co (Bowes et al., 2012b). This panel is composed of 48 proteins that we call Very Important Targets (VITs). We positioned the VITs in the interactome and calculated the shortest path distance of each one of the proteins considered in our training set to any VITs using NetworkX (Ceol et al., 2009). Of the overall distribution of shortest path distances to VITs of any given protein, the value of the first quartile was considered. This value represents the relative position of the given protein with respect to the VITs panel.

### 2.4 Model Construction

#### 2.4.1 Positive and Negative Sets

The positive set, i.e., proteins related to a given ADR, for each of the 84 ADRs considered were extracted from the T-ARDIS database (Galletti et al., 2021). For the purpose of training and since the number of positive cases per ADR was generally low, the positive set was augmented using the definition of close connectivity as follows. The DIAMOnD score (Drozdetskiy et al., 2015) was computed for the subnetworks associated with the ADR’s associated proteins extracted from T-ARDIS. In doing so, we ranked the most immediate neighboring proteins and selected those with a DIAMOnD score over a certain threshold to conform to the positive set. Also, multiple DIAMOnD threshold scores have been tested to obtain the best result during the training phase, namely, at 0.6, 0.7, 0.8, and 0.9. Likely, the negative sets were specific to each of the ADRs under consideration by randomly selecting proteins with a DIAMOnD score below the given positive threshold. During the training and testing phase, different ratios of positive and negative cases were tested to account for class imbalance. Indeed, besides using a balanced training set, i.e., equal number of positive and negative cases, to train and test the models, different ratios including 1:1.5, 1:3, and 1:5 (positives:negatives) were also considered. Thus, in the end, for each one of the 84 ADRs, 12 different models have been obtained by the combination of positive and negative thresholds as well as imbalance ratios resulting in 1,008 trained models.

#### 2.4.2 Features Vectorization and Model Construction and Training

**FIGURE 1:**
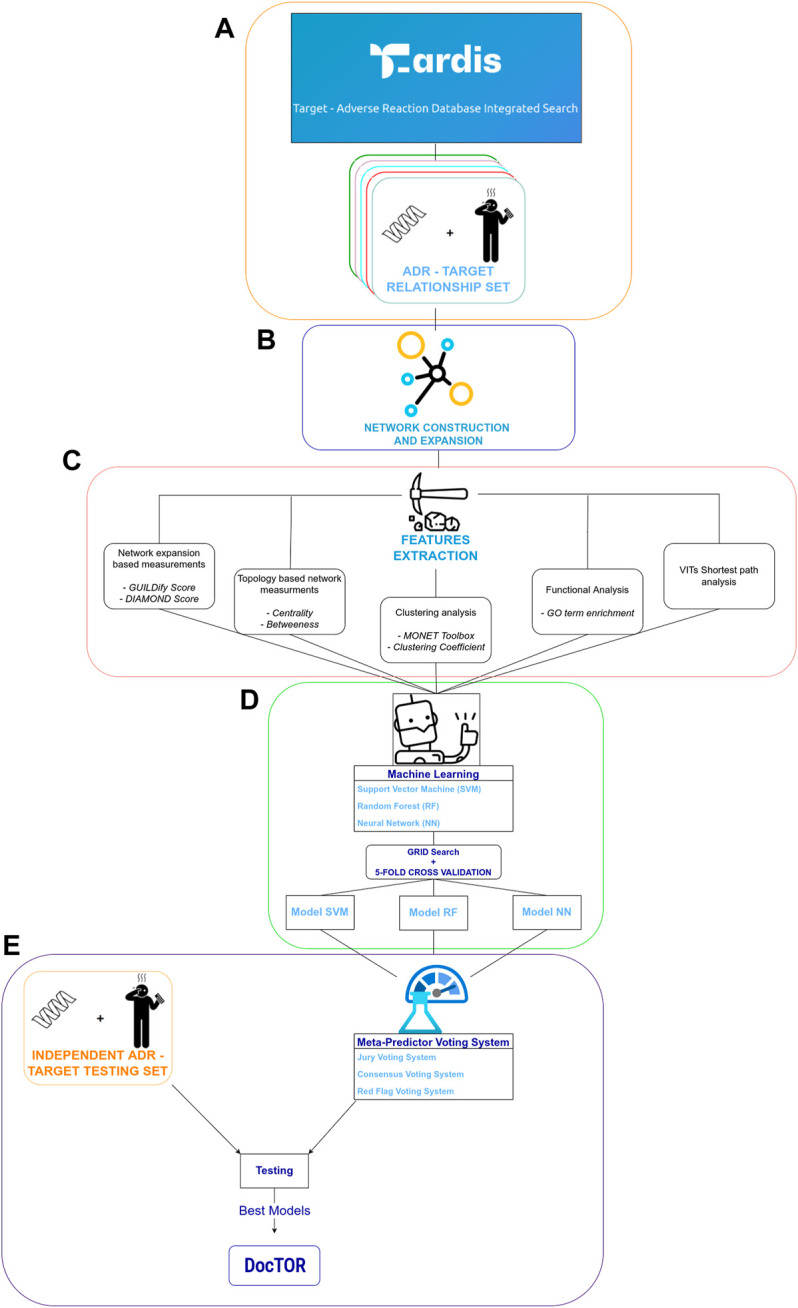
Schematic depiction of feature extraction, training, and testing procedures. **(A)** indicates the process of extraction of training dataset from T-ARDIS (Galletti et al., 2021). **(B)** indicates the process of network expansion of targets extracted in **(A)** using GUILDify (Aguirre-Plans et al., 2019). **(C)** summarizes the process of computation of different input features. **(D)** Represents the development of machine-learning classifiers. Finally, **(E)** illustrates the development of the meta-predictors together with the testing of the classifiers and consensus functions on the independent dataset.

The approach to predict protein–ADR associations is described below. In a nutshell, the approach is network-based, i.e., relies on a network-based set of 8 metrics computed for each protein that were used as inputs to machine-learning classifiers. Three different types of classifiers were used: SVM with nonlinear kernel (radial basis function—RBF), RF, and NN. The different ML classifiers were implemented in python3.9 using the following libraries. SVM and RF classifiers were implemented using the *Scikit-learn* package (Pedregosa et al., 2011), while NN made use of the Keras and Tensorflow packages (Abadi et al.,2015; Gaulton et al., 2017). Specific models were trained and tested for each of the 84 ADR as well as models at SOC, i.e., grouping ADRs belonging to the same SOC. A schematic representation of the overall process is depicted in Figure 1.

Each protein in a given ADR is represented by an 8-dimensional vector composed by the features described above (or see Figure 1) that is used as an input to the classifier together with the labels (positive/negative) in supervised learning. Note that balanced and unbalanced sets were used, and thus, 4 specific models were built for each ADR depending on the set used. The training involved the optimization of a set of parameters using a grid-search approach and validated with an internal stratified five-fold cross-validation approach using the Scikit-learn python package. In the case of SVM classifiers, the grid search included the *gamma* and *C* parameters; for the RF, the *maximum number of features* and the *depth* for each tree; lastly, for the basic model architecture of NN, an *SGD optimizer function* was combined with a *relu activation function* (for the first layer) and then with a simple *sigmoid activation function*. A grid search was used to optimize the *learning rate, number of epochs*, *number of hidden layers*, and *neurons*, the same as it was for the other ML algorithms. Finally, in the case of ML classifiers derived for SOC, i.e., groups of ADRs, the training and testing was done in the same way after merging all the elements in each individual ADR. The training dataset, including the ML classifiers for individual ADRs and SOCs, can be obtained from https://github.com/cristian931/DocTOR together with the relative parameters of the best model for each ADR (Supplementary Material—NN_parameters.tsv, RF_parameters.tsv, SVM_parameters.tsv).

### 2.5 Assessing Performance of Models

The performance of models was assessed using four widely used statistical descriptors, namely, the accuracy (ACC), precision (PREC), recall (REC), and MCC calculated using the Scikit-learn python package (Pedregosa et al., 2011). In addition, the scores of AUPRC have been computed and compared to the NPV and PPV values available in the Supplementary Material S1.

### 2.6 Combining Predictions: Voting Systems

Three different voting systems were envisaged to integrate the prediction of individual classifiers: a *jury vote*, a *consensus* score, and a *red-flag* schema. Both jury votes and consensus seek to maximize similar predictions, while the *red-flag* prioritizes outliers. Jury voting is simply the count of prediction outcomes. Classifiers are binary and thus will predict whether a given protein is or is not causing a given ADRs. Each method exhibits a vote, and the most voted option is selected. The consensus score *c is* more granular, namely instead of a yes/no the posterior probability *p* of each classifier is used. Therefore, the consensus score can rank proteins within the same class, e.g., predicted to be related to a given ADR. Finally, the *red-flag* schema simply accepts as a final prediction the one which is not common among the different classifiers.

$$c=\overset{3}{\underset{i=1}{\sum }}p_{i}*class\left(i\right);i=\left[SVM,RF,NN\right];class\in \left[-1,+1\right]$$ 1

## 3 Results

### 3.1 Individual Features

**FIGURE 2:**
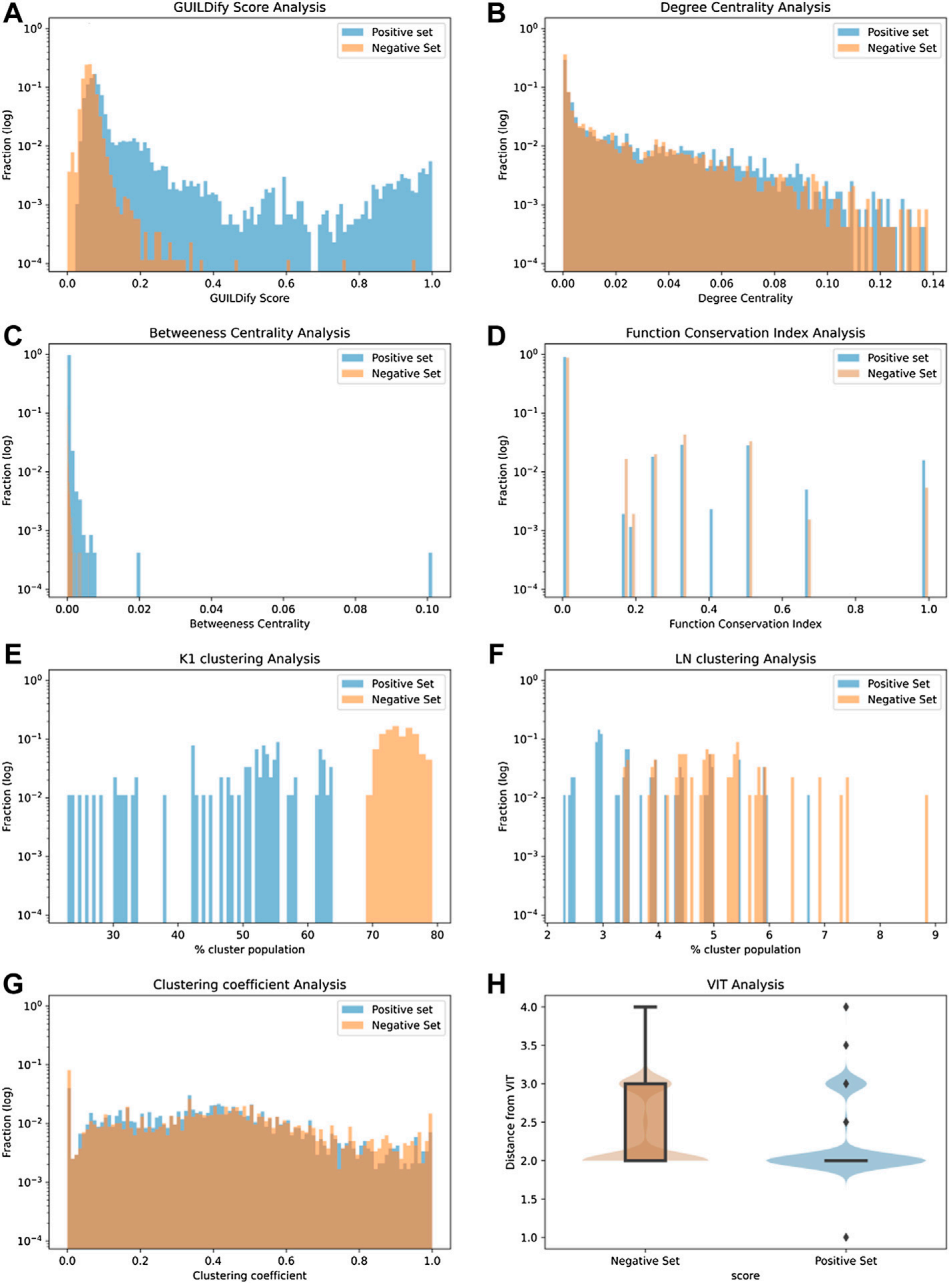
Distribution plots of 8 different input variables used by classifiers. The values of the positive and negative sets are shown in blue and red, respectively, in **(A–G)** and shows the distribution of GUILDify scores, centrality values, betweenness values, function score, % of clusters K1, % of clusters LN, and clustering coefficient values respectively. **(H)** presents the box-plots and a violin representation of the distribution of the shortest path values on the negative (orange) and positive (blue) sets.

**FIGURE 3:**
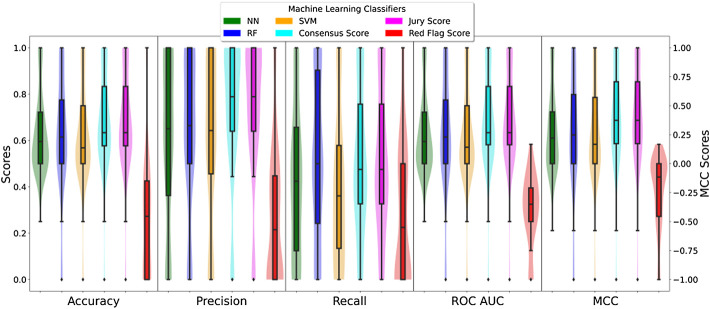
Box- and violin plots of the cross-validation AUC results for the three different classifiers. The different box-plots show the distribution of the mean AUC values for the best models developed for each ADR using the three different classifiers: SVM (orange), random forest (blue), and neural networks (green).

**FIGURE 4:**
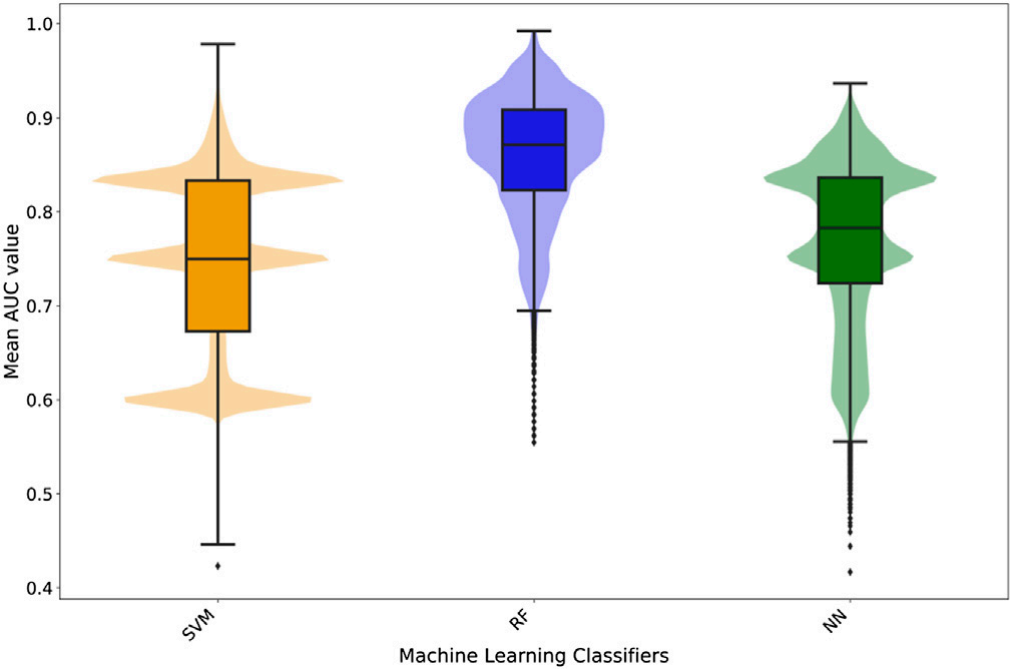
Box- and violin plots for accuracy (ACC), precision (PREC), recall (REC), receiver operating area under curve (ROC AUC), and Matthew correlation coefficient (MCC). Distribution of accuracy, precision, recall, and ROC AUC values for individual classifiers: NN (green), RF (blue), and SVM (orange) as well as meta-predictions: *consensus* (cyan), *jury vote* (magenta), and *red flag* (red).

Eight different variables were considered as input features of the classifiers. These include the GUILDify scores, network topology (degree and betweenness centrality values), a function conservation score, module imputations, and distances to proteins belonging to safety panels. In Figure 2, the distribution of the different features for the positive and negative sets is shown. As mentioned in the Methods section, the positive cases (negative cases were selected randomly) were extracted from the T-ARDIS database (Galletti et al., 2021), both for the self-reporting and curated sets. The data shown in Figure 2 derives from the self-reporting set of T-ARDIS. The equivalent information for the curated set is shown in Supplementary Figure S1; Supplementary Material S1. Likewise, equivalent information, as in Figures 3, 4, is presented in the Supplementary Material S1.

In the case of GUILDify scores, a high overlap is found, but nonetheless, the positive sets demonstrate higher scores and a distribution slightly skewed toward high values (Figure 2A). The analysis of centrality-based features also indicates a substantial overlap between positive and negative sets, although positive sets present a more skewed distribution toward higher values particularly in the case of betweenness values (Figures 2B,C). A similar situation is presented when a quantifying function analysis as distance to enriched function(s) of the set (Figure 2D); the proteins in the negative set tend to demonstrate larger distances, i.e., no shared functions with the GUILDify enriched GO terms, respect to those on the positive set. In fact, the largest number of proteins with a value of 1.0 correspond to the proteins in the positive set and, conversely, those with lower values, i.e., no shared GO terms, tend to be proteins in the negative set. However, it is fair to say that the overlap is very high.

The tendency of functionally and disease-related proteins to be close (i.e., shorter distances) in the interactome was also considered as a feature for the prediction. As described in the Methods section, this aspect was studied by applying clustering algorithms to identify modules in the entire interactome where the proteins associated with the same or similar ADRs are grouped. Next, if the number of modules required to represent a given collection of proteins in an ADR is small, it is likely that the proteins will share modules. Similarly, a large number of modules indicate that the proteins do not share the same cluster. The K1 algorithm (Cao et al., 2014) identified 1,170 different clusters, many of them composed of 3 proteins, the least amount for defining a module (Figure 2E). As shown, proteins in the positive set present a lower number of clusters, meaning that proteins associated with ADRs tend to belong to a limited group of clusters, rather than being scattered through the interactome. Similarly, the Louvain-Newman method (Blondel et al., 2008), which grouped the whole interactome into only 95 distinct clusters, allowing the analysis of bigger modules, demonstrated a similar distribution as K1, i.e., the positive set is drawn toward lower values (Figure 2F). Finally, in the case of the Clustering Coefficient Analysis (Figure 2G), in this case, both negative and positive sets share the same distribution of values. Therefore, this feature does not seem to provide a clear distinction between positive and negative cases on the ADR.

The final metric considered as an input variable was the distance of given proteins to the so-called VITs (see Methods). The distance was computed in the form of the shortest path (i.e., lowest number of links) to any given protein belonging to the panel, taking the value of the first quartile upon computing all the distances all vs. all (protein in the given ADR and proteins in the panel). Once again, the distribution of values is different depending if the proteins are part of the positive or negative sets (Figure 2H). While the most common distance is 2.0, only the proteins in the positive set would demonstrate values smaller than 2, therefore showing that proteins in the positive set are closer to proteins considered critical as per pharmacological profiling.

### 3.2 Training and Cross-Validation

The input features described above represent the input variables to the different classifiers explored in this work. Three different machine-learning methods were used: NN, SVM, and RF. In order to define the best parameter values, each classifier was trained and validated on a 5-fold cross-validation and grid-search approach.

It is important to mention that specific classifiers were developed for each ADRs. The classifiers are not generic predictors of the likelihood of a protein to elicit an ADR, any, but to elicit a particular ADR, e.g., diarrhoea. Therefore, the predictions are tailored to the specific ADR (84 considered in this study) and, therefore, present unique characteristics. Next, Figure 3 presents the distribution of mean area under the ROC curve (AUC) calculated for the training and testing as described (for details on individual classifiers and ADRs refer to the Supplementary Material S1—Supporting information 7 “cv scores. zip”). In general RF classifiers appear to demonstrate higher performance with mean AUC values around 0.85. Also, RF presents a more bell-shaped distribution of values when compared to SVM and RF. On the other hand, SVM and NN demonstrate a comparable performance, with a median AUC around 0.75, although the first quartile in SVM is slightly better than in NN (0.72 vs. 0.68).

Overall RF appeared to demonstrate the best performance under training conditions, but in some cases, the performance of the different classifiers was lower for particular ADRs, highlighting the complexity and heterogeneity of this biological problem. For instance, in the case of the ADR *malnutrition*, RF achieved the best performance with an accuracy, precision, recall, and MCC values of 0.95, 0.92, 1.00, and 0.91, respectively. However, in the case of the ADR *febrile neutropenia*, NN was by far the best predictor with an accuracy, precision, recall, and MCC values of 0.80, 0.87, 0.70, and 0.77, respectively, against an almost random prediction by SVM and RF (MCC ∼0.0). Finally, SVM outperformed the other two ML approaches in other cases, such as *Nasal Congestion*, with an accuracy of 0.90, a precision of 0.83, a recall of 1, and a MCC of 0.81, while RF and NN barely reached values of 0.70 (see Supplementary Material S1 for detailed information of individual performances across all ADR studied).

### 3.3 Testing on Independent Set

For independent testing purposes, we relied on proteins associated with the same ADRs retrieved from external sources, as described in the Methods section. This testing set is formed of 188 different proteins associated with 84 ADRs. Also, the training and the testing set do not overlap, meaning none of the 188 proteins present in the test set were present in the training set. The proteins associated with each one of the 84 ADRs are predicted using the respective model, and then, the performance score is computed based on the results (Figure 4).

Very large differences were not found between the different classifiers. They appear to perform at a comparable level in terms of accuracy, precision, and AUC, although RF appeared to achieve a higher performance particularly in the case of sensitivity with the highest value for the 3rd quartile of the distribution. In terms of MCC, values are distributed mainly above 0 values with the median values around 0.25, thus indicating non-random predictions (Figure 4).

### 3.4 Combining Predictors

Since three different classifiers were developed for each ADR, the possibility exists of combining the predictions using consensus scoring functions. Three different approaches were used as described in Methods. In terms of accuracy, precision, recall, and AUC, the values increased when compared to individual predictors in the *jury vote* and *consensus* voting systems (Figure 4). There was not only an improvement but also a general shift toward higher values as distributions were skewed toward higher values. The exception was the *red-flag* consensus that resulted in a worsening of predictions. As described in the Methods section, the *red-flag* method was devised to identify singular predictions.

A similar pattern is observed in the case of MCC values (Figure 4). The distribution of MCC values for *jury vote* and *consensus* voting systems were skewed toward higher values when compared with individual predictors. Thus, the quality of the prediction improved when combining individual predictors. As shown in the of accuracy, precision, and recall, *red-flag* consensus decreased resulted in worse MCC values distributing between 0 (random prediction) and negative (inverse) values. Therefore, it is a better strategy to accept the most common prediction rather than any singular predictor.

### 3.5 Predicting at SOC Level

**FIGURE 5:**
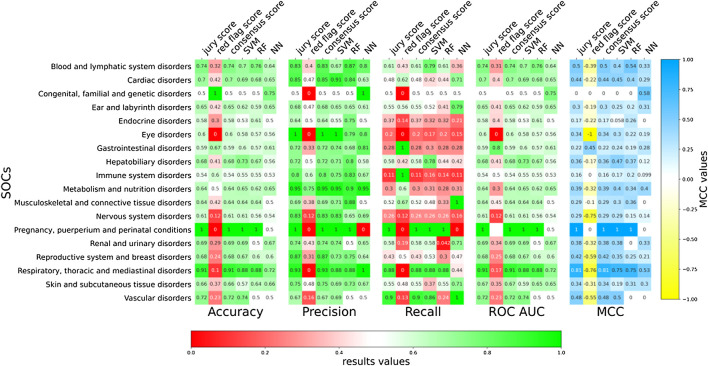
Evaluation of ADR-protein association predictions of the different classifiers at SOCs level. Accuracy, precision, recall, and ROC AUC values for predictions at SOCs for both individual classifiers (SVM, RF, and NN) and voting (*jury vote*, *consensus*, and *red flag*).

The models presented in the previous sections were ADR-specific. However, we also wanted to develop more generalist predictive models that at the same time preserve the biological and medical meaning. For this purpose, we grouped the different ADRs into specific SOCs as per MEDDRA classification (Chang et al., 2017). The MedDRA SOC is defined as the highest level of the MedDRA terminology, distinguished by anatomical or physiological system, aetiology (disease origin), or purpose. Also, most of these describe disorders of a specific part of the body. As explained in the T-ARDIS manuscript (Galletti et al., 2021), not every SOC is present in the database due the fact that some MEDDRA reported ADRs are very general or not specific to body parts, tissues, or underlying human biology (Ietswaart et al., 2020). Specifically, in this study, the 84 ADRs considered were grouped into 18 different SOCs with an average number of 5 ADRs per SOC. At a single classifier level, a large variability of predictions was found in terms of accuracy, precision, sensitivity, and MCC (Figure 5). Predictions were highly accurate in the cases of “*pregnancy, puerperium, and perinatal conditions*” compared to those in the case of *immune* or *nervous* disorders. In general, combining predictors resulted in improved predictions, with the exception of *red-flag* voting, particularly in terms of recall. However, sensitivity values were generally low when compared to those achieved by predictors working at ADR level (Figure 4). This fact highlights the difficulty of predicting at a higher level of abstraction rather than at individual ADR level.

In terms of MCC values, a similar situation can be observed (Figure 5). There was an improvement of predictions when combining individual prediction in a *jury vote* or *consensus* voting, such in the case of *respiratory, thoracic, and mediastinal disorders* going from a MCC of 0.75 of the best predictor to 0.81 when combining.

## 4 Discussion

In this work, we set to develop an approach to predict the potential liability of proteins in the context of adverse reactions when targeted for therapeutic purposes. By analyzing the human interactome, a range of network-based metrics were derived to characterize the proteins under study. This range of heterogeneous measurements was then fed into three machine-learning classifiers that were in turn combined using three different voting approaches. The prediction models both at individual ADRs and SOCs level provided a reasonable performance that justified its use as a tool to foresee potential liabilities of proteins. We looked at 84 different ADR in total, being able to create reliable models for each of them.

### 4.1 Classifiers Performances

The variables used in the predictions were of eight accounting for different aspects of the proteins under study. As shown in Figure 3 , the level of discrimination among positive and negative cases varies with GUILDify scores and K1 clustering analyses among the top performers and degree centrality and clustering coefficient analyses as fewer discriminating features. This reflects the small world nature of the human interactome (Zhang and Zhang, 2009). As shown in the results, the performance of the different classifiers varied, with RF being the overall best performed predictor under training conditions, although in particular, ADRs, SVM, and NN were superior. This observation prompted us to develop a voting system to combine the individual predictors in a meta-predictor fashion. As shown in Figures 4, 5, combining the methods resulted in better predictions with the exception of the *red-flag* consensus. Both the *jury vote* and *consensus* voting systems followed the same principle, i.e., to boost coincident predictions among classifiers. In fact, the level of performance of jury *vote* and *consensus* voting systems are comparable (Figures 4, 5), but critically, the *consensus voting system* provides further granularity to the predictions that allows a finer ranking. Indeed; however, for instance, a *jury vote* will place a given protein in a class, e.g., +1; the two methods will agree that the given protein might be linked to a given ADR, and the *consensus* scoring function, however, will provide a quantitative measure that can allow the ranking of proteins within the same class. This aspect is pivotal in order to establish a degree of confidence in the predictions of the DocTOR application (see below). Finally, as mentioned, the *red-flag* voting system resulted in worse predictions overall. The idea in itself seems counter-intuitive, i.e., promoting the marginal view. However, a few cases are found where this strategy was successful such in the cases of *nocturia, neutropenia,* or *ischaemia* ADR (see Supplementary Figure S1. tsv or Supplementary Figure S2. tsv). Furthermore, the *red-flag* approach serves as a failsafe in the event of an unknown prediction, such as in the instance of the DocTOR utility (explained below), or while two ML approaches, while agreeing, report low probabilities in their respective predictions.

The other aspect to consider in this work was the nature of the predictions. In theory, one of the major achievements of protein–ADR predictions would be determining if targeting a protein would result in an unwanted adverse response, i.e., ADR. However, this is a very difficult question to turn into a predictive model, as the types of ADR are very diverse, and we might end up considering any protein susceptible to causing an ADR to a certain extent. This is the reason why the predictive models were ADR-specific, so that the prediction is not whether a protein might cause an undesired reaction, but what type of adverse reaction. However, grouping ADRs into common SOCs is possible. In doing so, individual ADRs are abstracted into a higher entity, and, thus, more generalist prediction models can be developed, i.e., a model to predict whether the targeting of a given protein can be associated to a specific SOC perturbation. As shown in Figures 5, 6, predicting at this level resulted in some SOCs demonstrating better prediction performances than others. SOCs with more defined affected tissues/organs tended to demonstrate better predictions that include more systemic representations. For instance, comparing predictions on the *respiratory, thoracic, and mediastinal disorders* vs. *immune system disorders* resulted in the former achieving better performances (accuracy: 0.90 vs. 0.54; precision: 0.93 vs. 0.87; recall: 0.87 vs. 0.10; MCC: 0.81 vs. 0.16). Finally, researchers also found that better performance at SOCs related to cases with models already predicted successfully at the individual ADRs included in the particular SOC.

### 4.2 Difficult to Predict Adverse Drug Reactions

On the other hand, given the complexity of the biological problem, some ADR results are harder to predict. In particular, the worst results have been obtained in 17 different ADRs which obtained a negative or equal to 0 MCC (random predictions). These includes *Hyper-coagulation, Ichthyosis, Coordination abnormal, Biliary cirrhosis, Acute hepatic failure, Hyper-ammonaemia, Azoospermia, Diplegia, Glucose tolerance impaired, Haemorrhagic diathesis, Hypoacusis, Ophthalmoplegia, Renal tubular acidosis, Hepatic failure, Coagulopathy, and Ischaemia.* Target on these ADRs included common genes (Supplementary Figure S6. tsv), such as TP53, 5HT1A, ACE, members of the CALM family, LEP, and IL8. In particular, these genes have been already annotated in T-ARDIS as targets with the highest number of associated ADRs (Galletti et al., 2021), thus partially explaining prediction’s inaccuracy.

### 4.3 The DocTOR Utility

The predictive models and accessory scripts to carry out the predictions as well as all the datasets employed in this study are available at the Direct fOreCast Target On Reaction (DocTOR) application available at https://github.com/cristian931/DocTOR. The application allows users to upload a list of proteins in the form of UNIPROT identification codes and a list of ADRs of interest (from the available models), in order to study the potential relationship between the two. The program will assign a positive or negative class to the protein output and a probability associated to the given class for all three different classifiers (SVM, NN, and RF) and voting systems (*jury vote*, *consensus,* and *red flag*). Users can, therefore, consider all this information when analyzing the prediction results. Also, the application lends itself to being easily updated, allowing the user to add new models for new ADR on request or retrain existing models when new protein targets are discovered to be associated with certain ADRs and/or given new releases of the T-ARDIS database.

## 5 Conclusion

Predicting associations between protein targets and ADR is desirable, particularly in preclinical drug development, in order to identify early in the process potential liabilities and toxicity-related aspects linked to proteins. In this study, we addressed this problem from an interactome-centric point of view. Next, we collected a range of protein features, including their topology characteristic in the human interactome, the spatial position related to specific *in vitro* validated ADR-related hotspots and their function associations. Also, we trained three different machine-learning approaches to construct models for 84 different ADRs, including a specific DILI related subset and 20 different SOCs using the various features. The models were optimized via grid-search and 5-fold cross-validations, and the results were tested in an independent dataset. The analysis of the performance of the models both under training and independent testing validated its use as a prospective computational tool, to assess the liability of proteins both at the level of specific ADR type and SOC. Finally, we provided access to the data, models, and predictive tools through a dedicated GitHub repository for the use of the scientific community. Researchers will be able to use the DocTOR utility in combination with *in vitro* investigations to assess the potential association between protein target modulation and the onset of ADR, reducing research time.

## Data Availability

The datasets presented in this study can be found in online repositories. The names of the repository/repositories and accession number(s) can be found in the article/Supplementary Material.
