# Supplementary figures and images for: Prediction of Adverse Drug Reaction Linked to Protein Targets Using Network-Based Information and Machine Learning
Source: Front Bioinform. 2022 Jul 14;2:906644. doi: 10.3389/fbinf.2022.906644 (PMC9580901; doi:10.3389/fbinf.2022.906644)

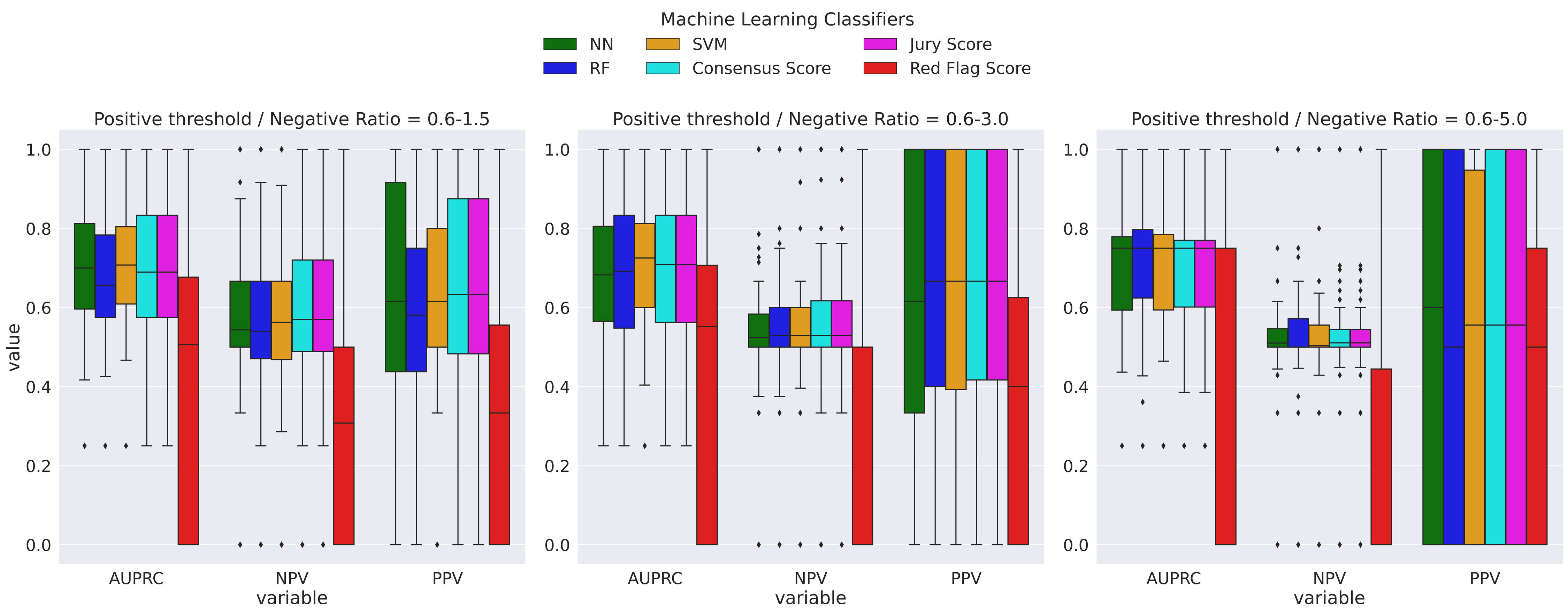

Supplement: Supplementary file 3 [file Image6.TIF]

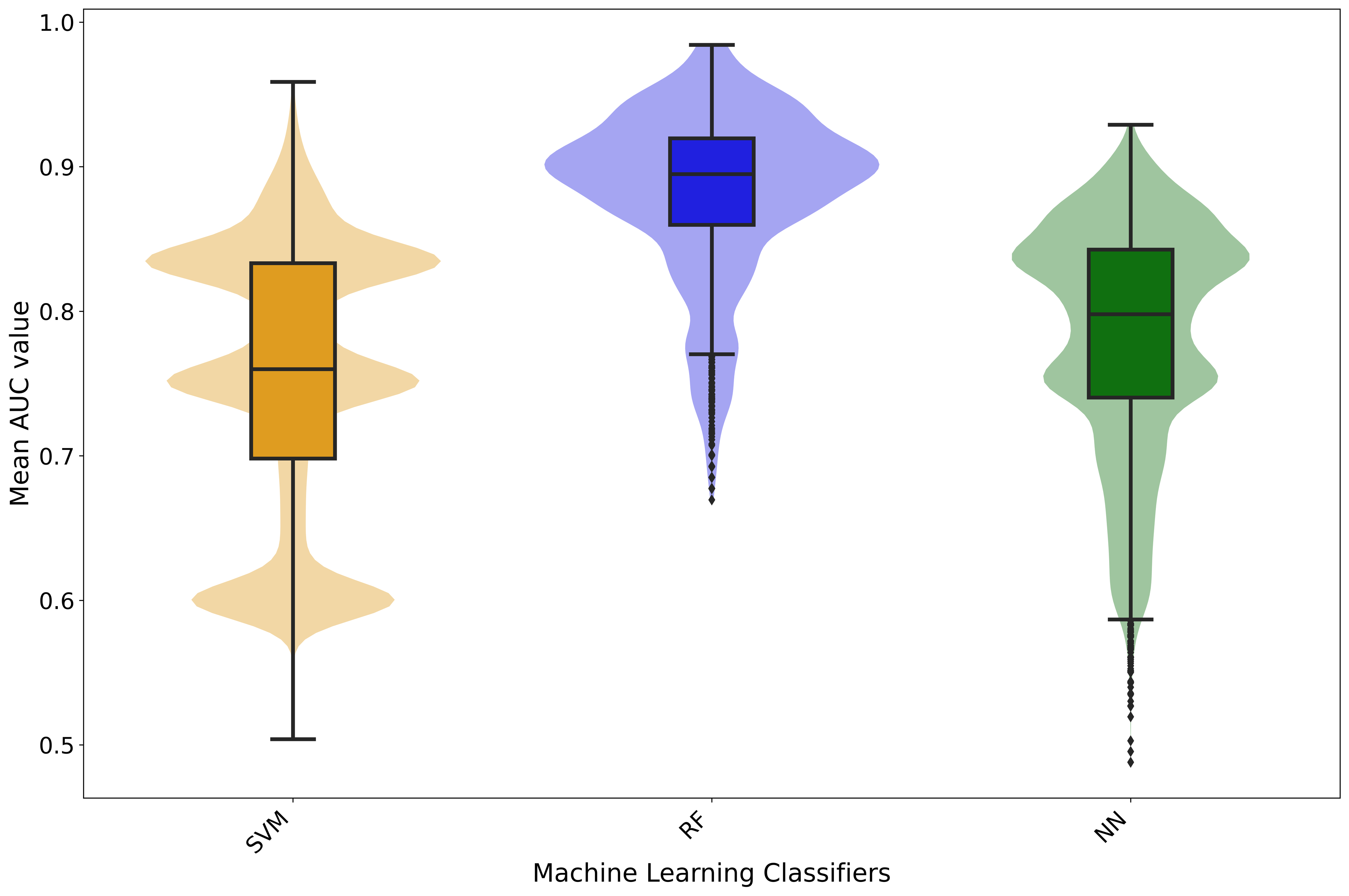

Supplement: Supplementary file 5 [file Image3.TIF]

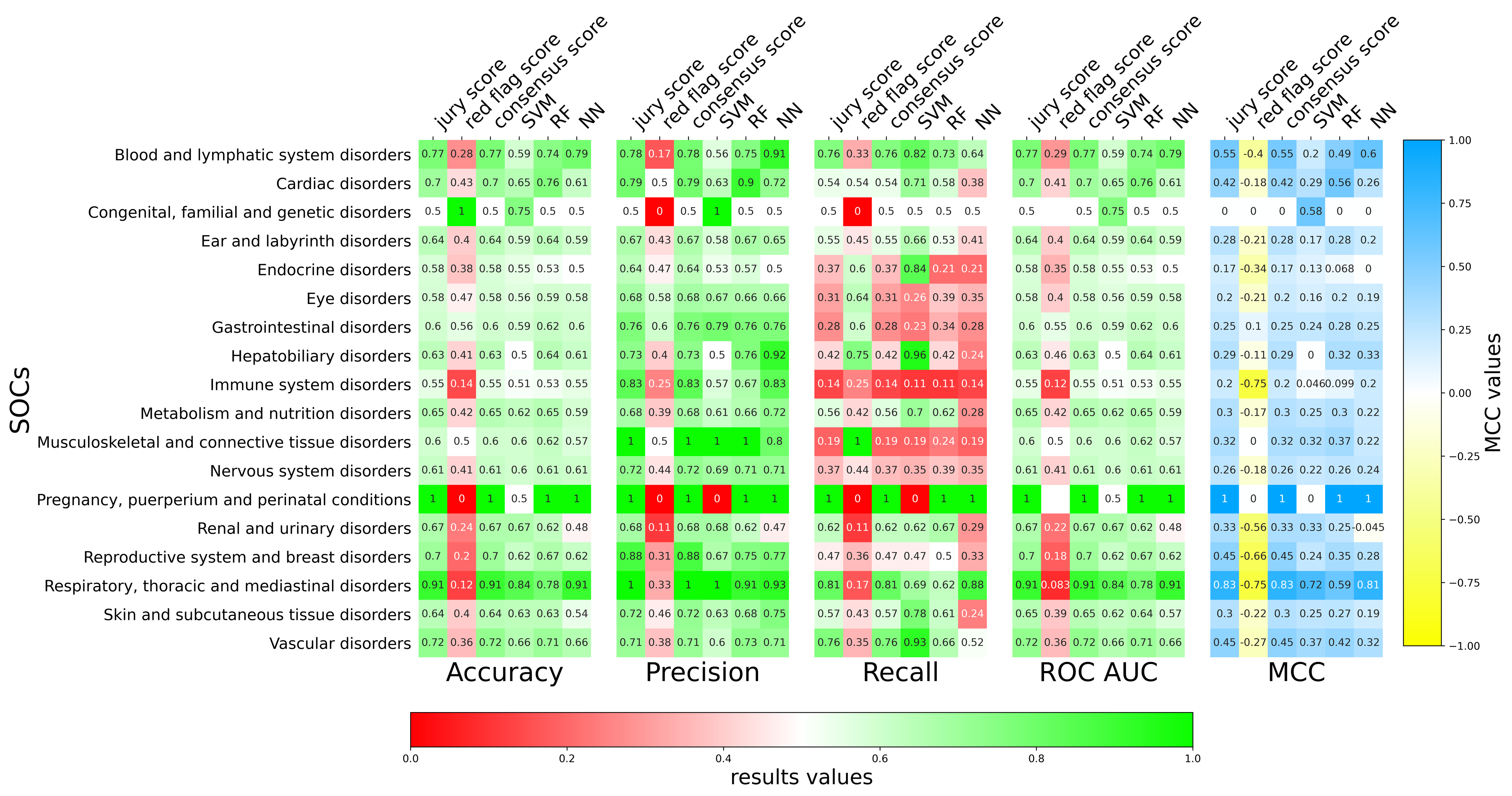

Supplement: Supplementary file 6 [file Image4.TIF]

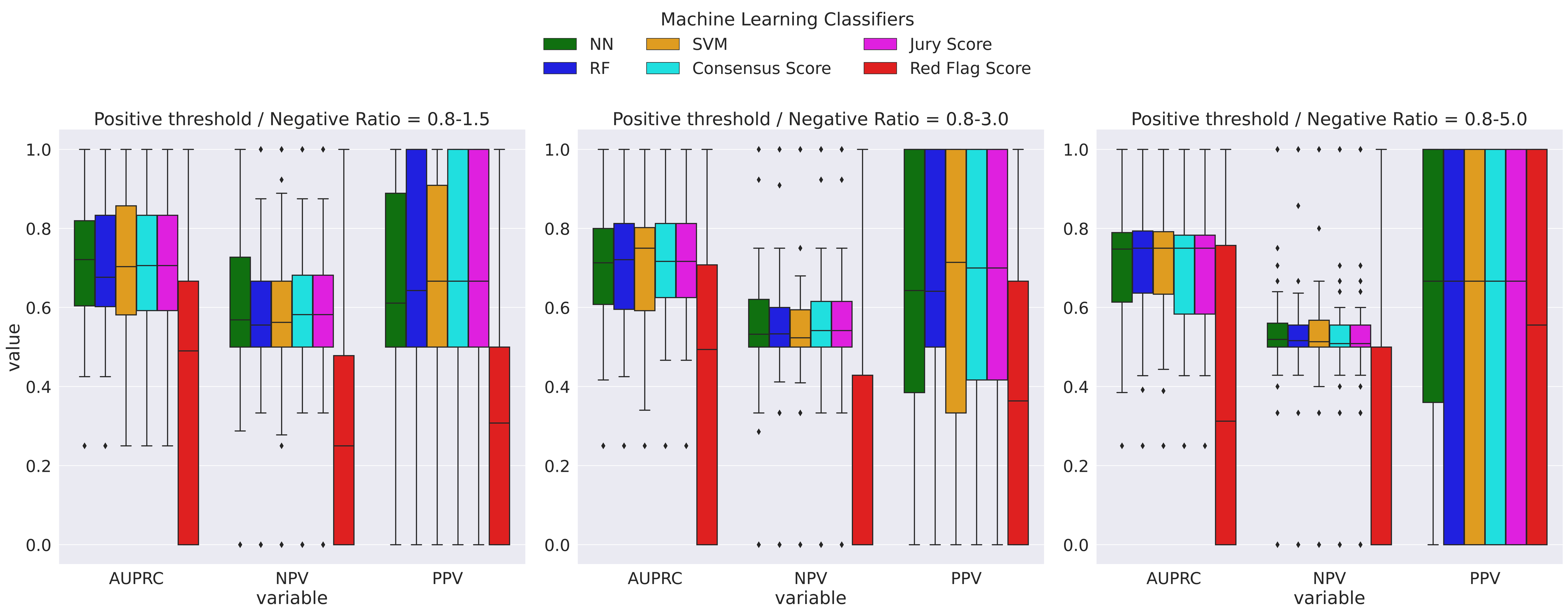

Supplement: Supplementary file 7 [file Image9.TIF]

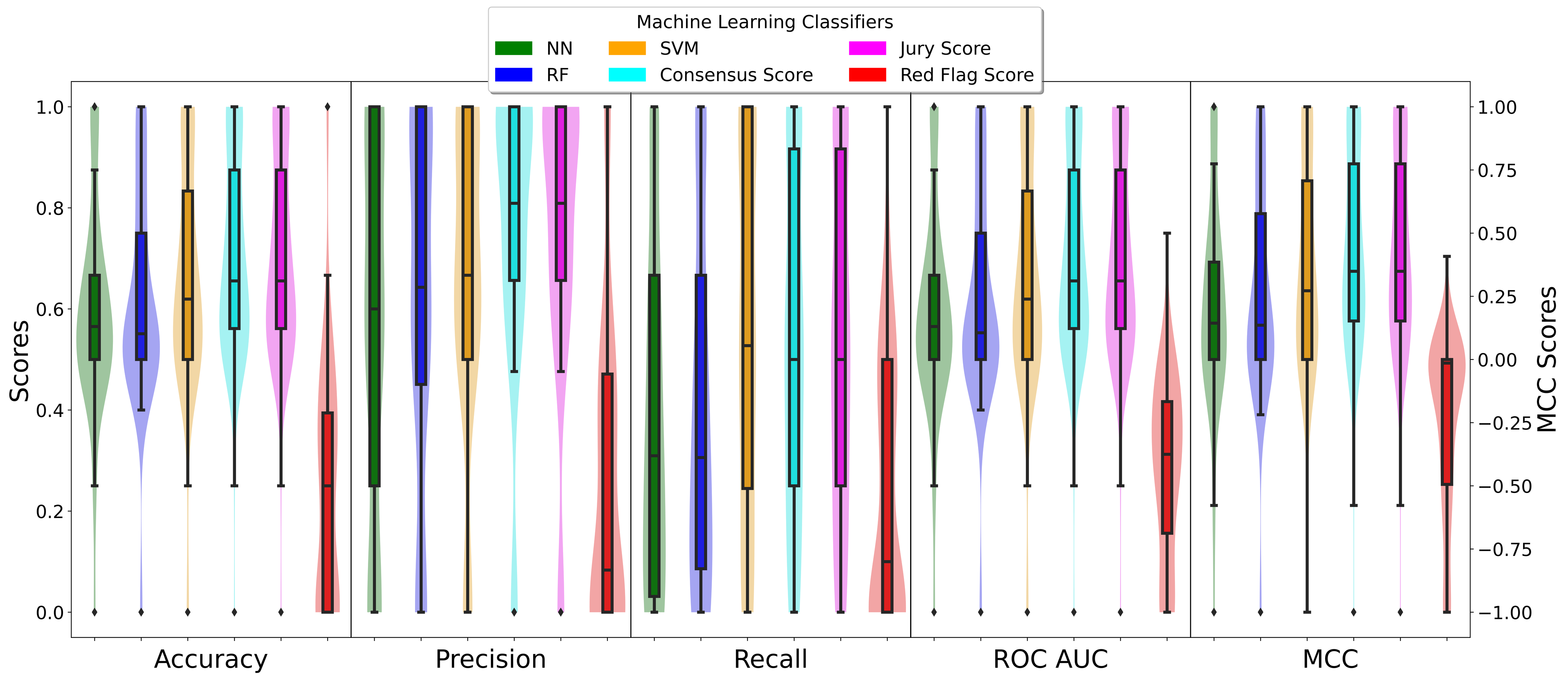

Supplement: Supplementary file 8 [file Image2.TIF]

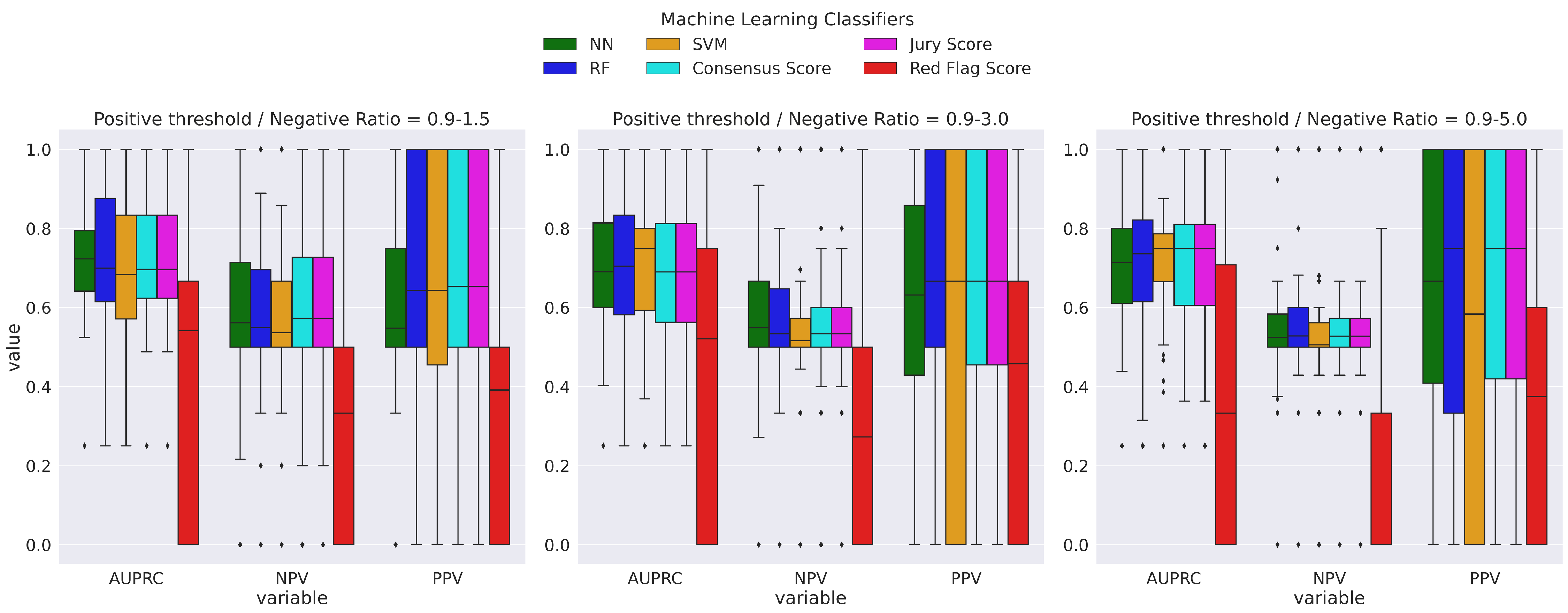

Supplement: Supplementary file 9 [file Image11.TIF]

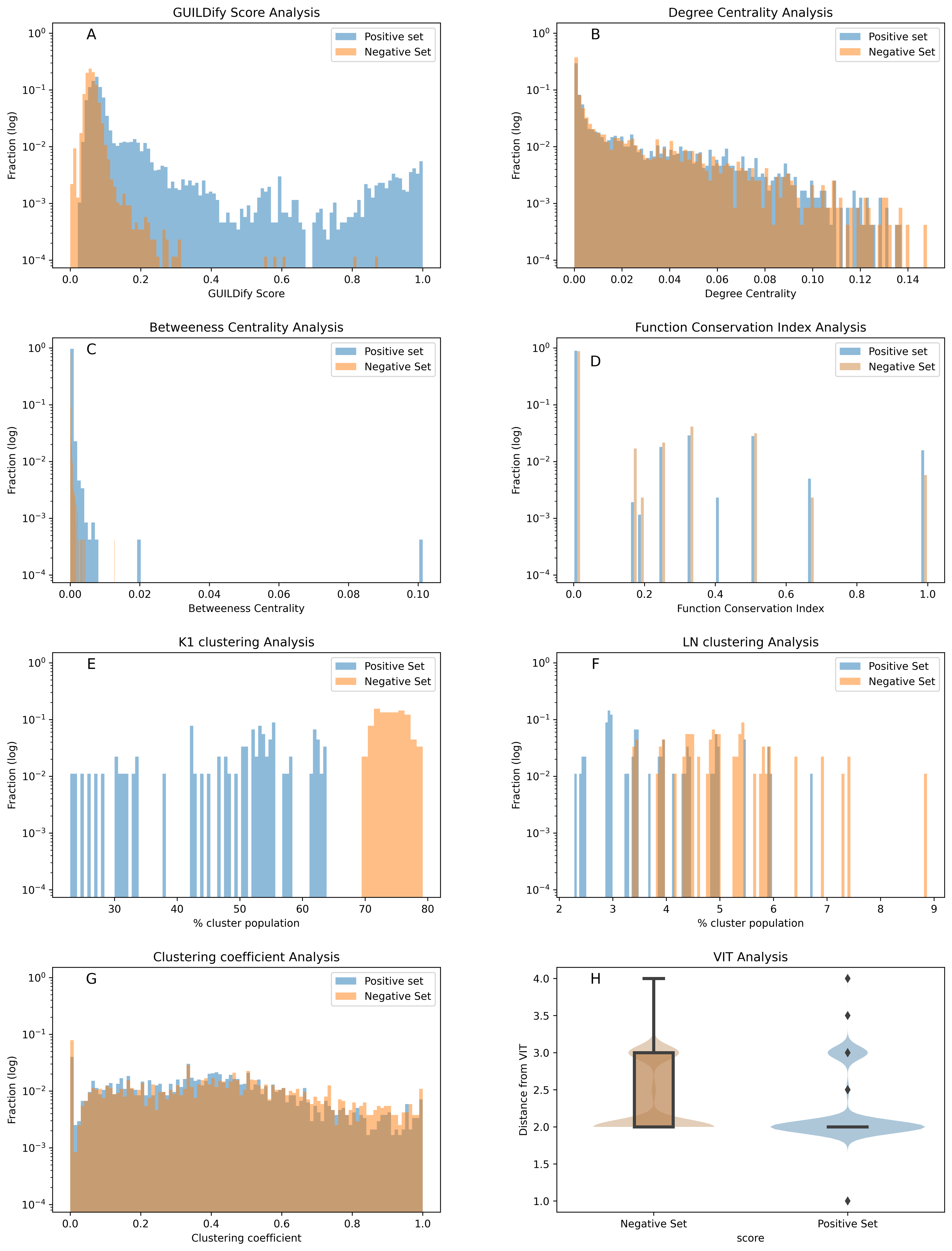

Supplement: Supplementary file 10 [file Image1.TIF]

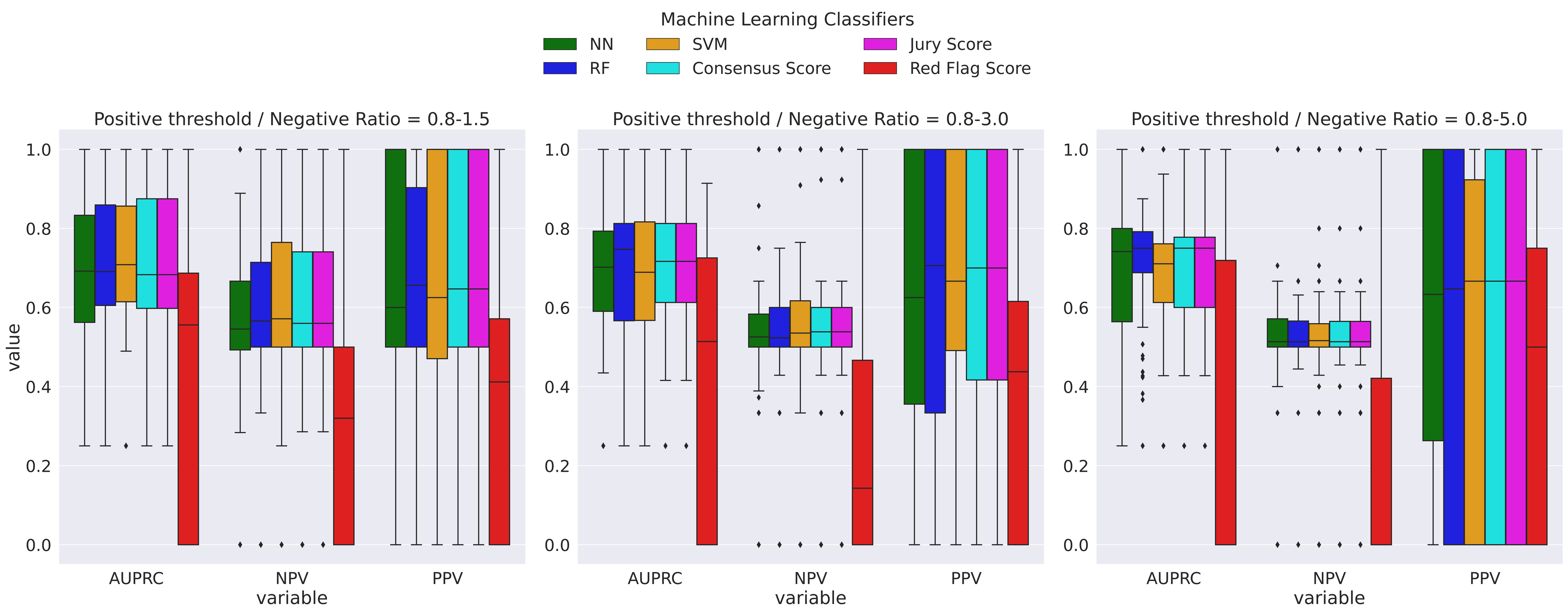

Supplement: Supplementary file 11 [file Image10.TIF]

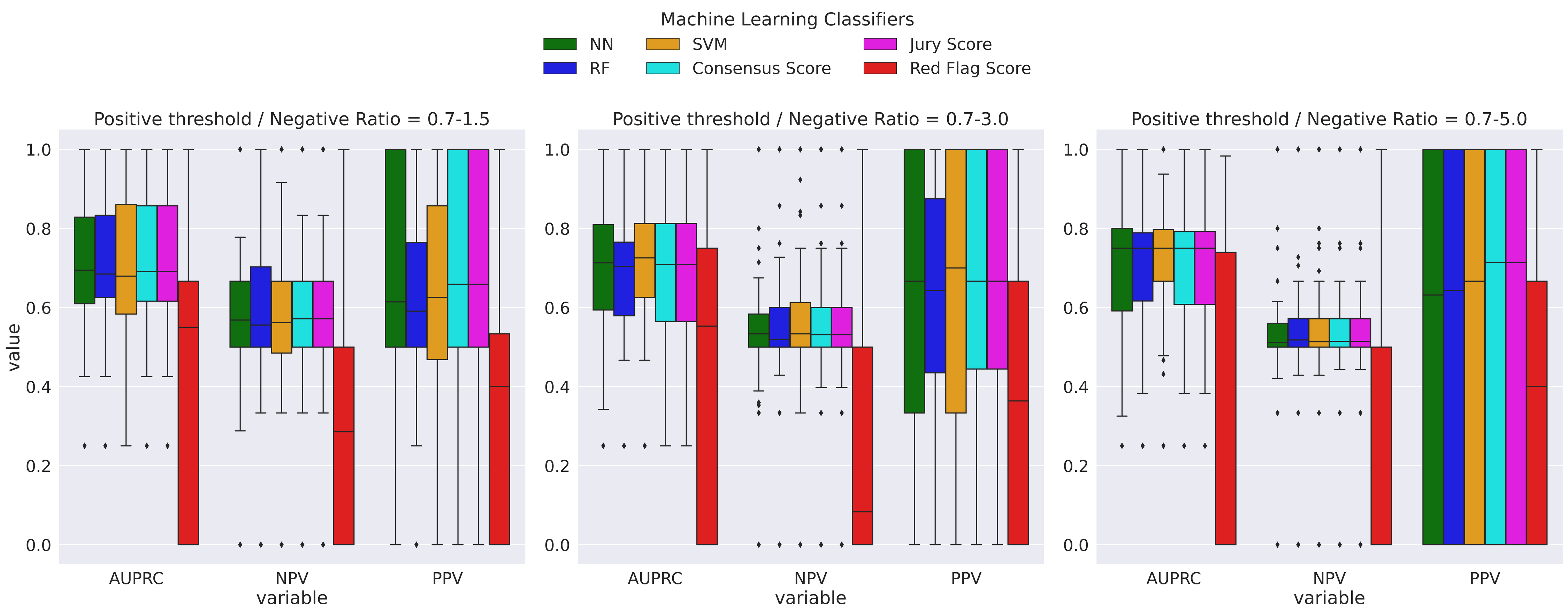

Supplement: Supplementary file 12 [file Image7.TIF]

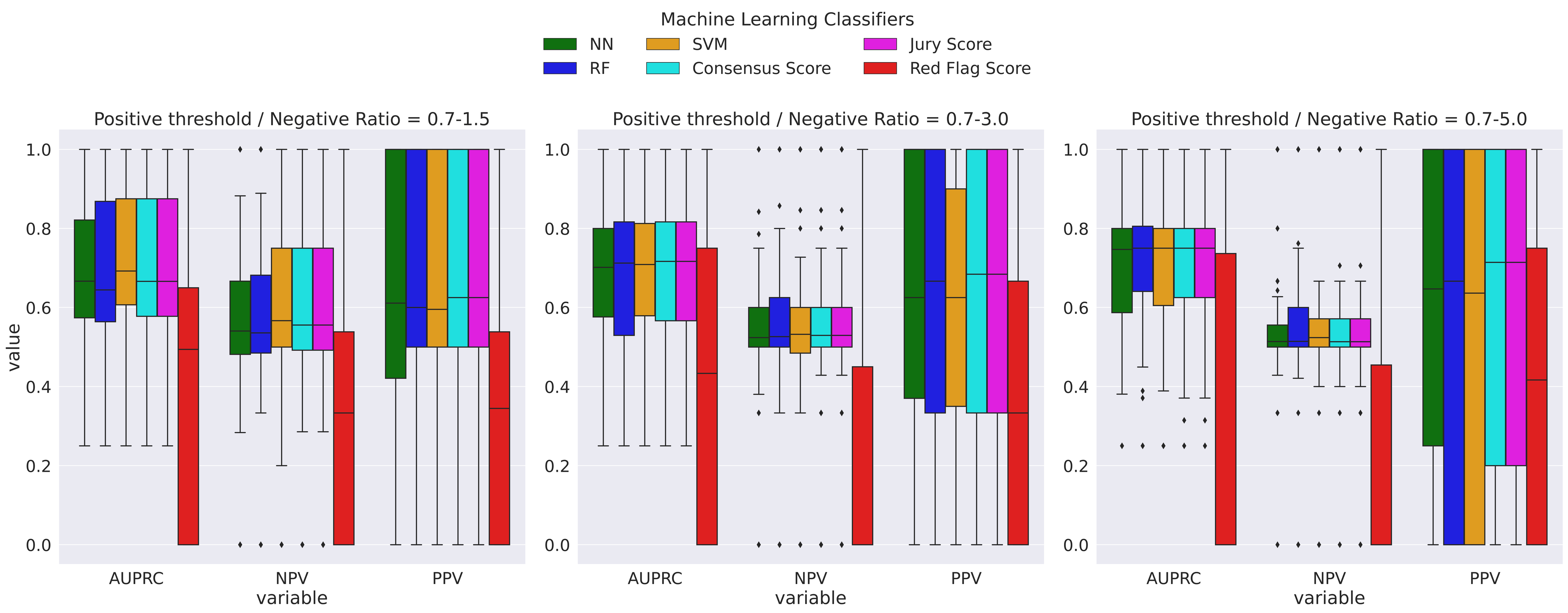

Supplement: Supplementary file 15 [file Image8.TIF]

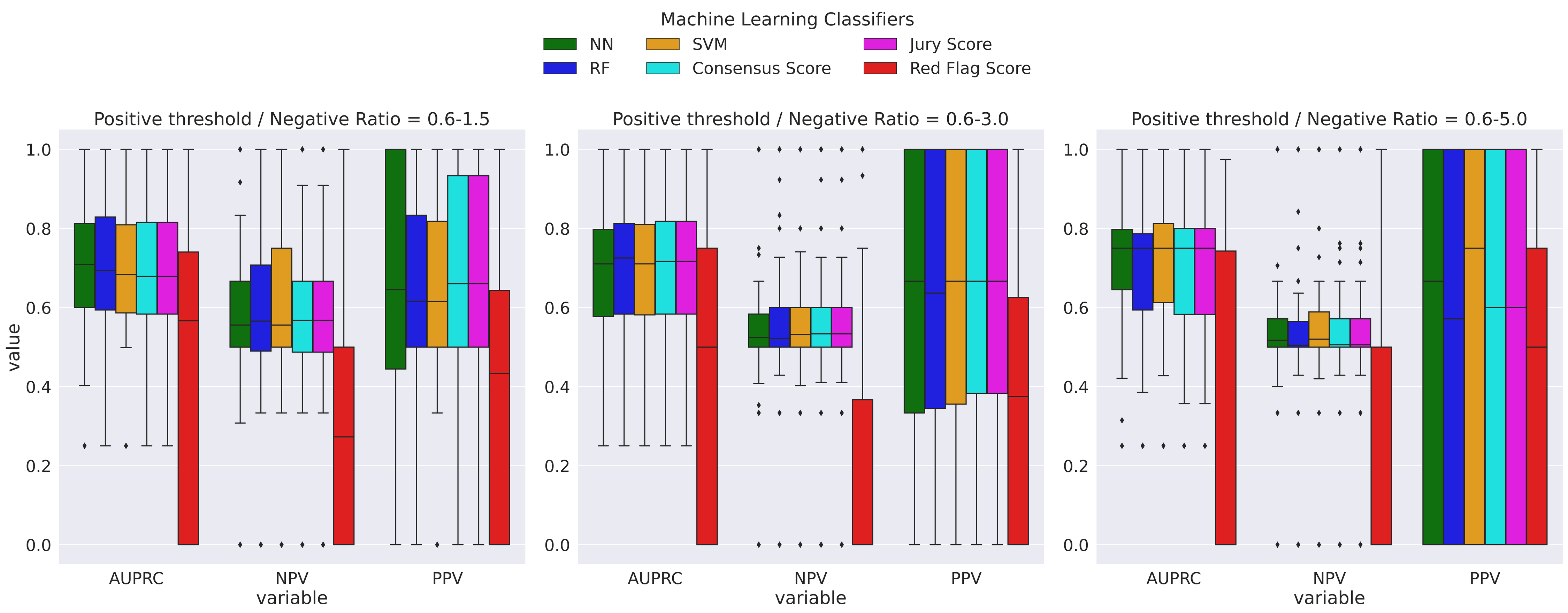

Supplement: Supplementary file 16 [file Image5.TIF]

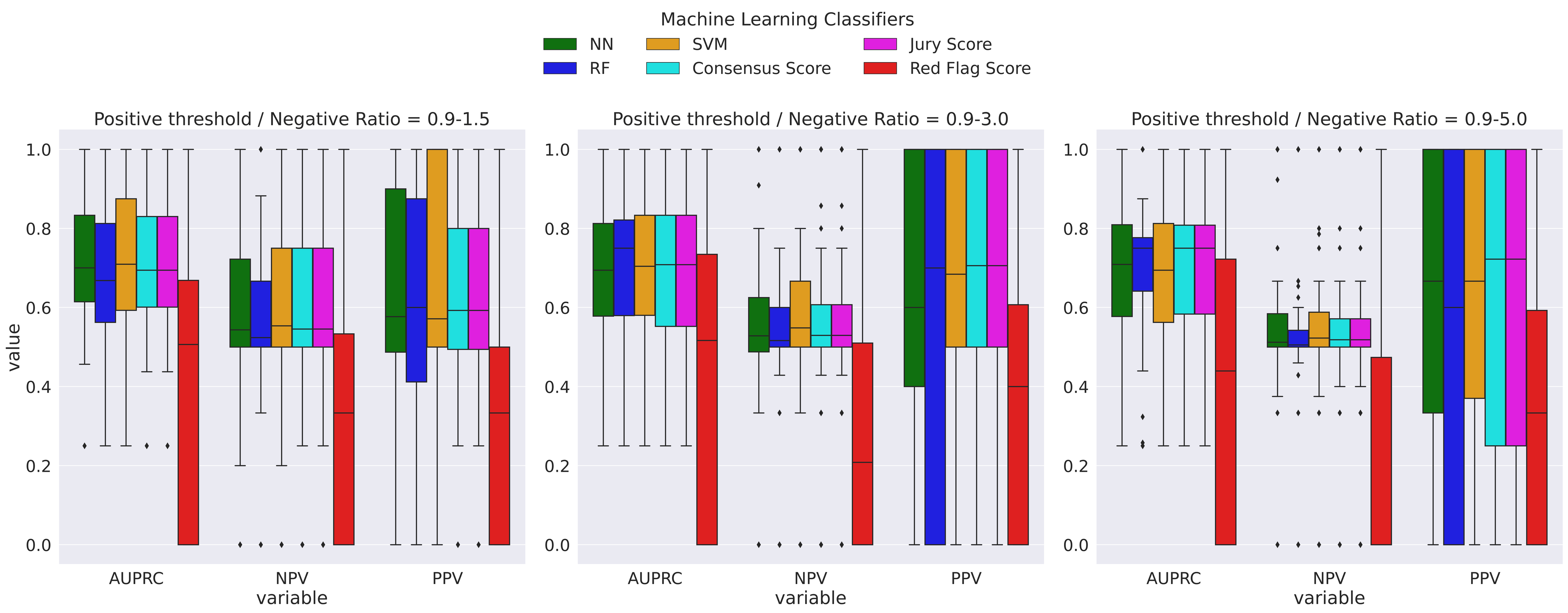

Supplement: Supplementary file 18 [file Image12.TIF]
