# Supplementary material for: Prediction of Adverse Drug Reaction Linked to Protein Targets Using Network-Based Information and Machine Learning
Source: Front Bioinform. 2022 Jul 14;2:906644. doi: 10.3389/fbinf.2022.906644 (PMC9580901; doi:10.3389/fbinf.2022.906644)
Supplement: Supplementary file 4 [file Table1.DOCX]

**SUPPLEMENTARY MATERIALS**

**Curated vs self-reporting set**

The ML techniques' distribution of scores reflects the varying number of proteins linked with ADRs in the self-reporting and curated T-ARDIS dataset.

Due to the nature of the origin databases, the curated set presents a more specific and trustworthy direct correlation between targets and ADRs, which is useful in the prediction of tissue-specific ADRs such as Atrial Fibrillation (curated jury score ACC 0.88, PREC 0.88, RECALL 0.88, MCC 0.77). However, this has an effect on the dimension of the GUILDify subnetwork and, as a result, on the definition of positive set during the training phase, resulting in somewhat poorer accuracy and precision for some difficult-to-predict associations such as *Respiratory Failure* ( *self reporting jury score* ACC 0.722222, PREC 0.7, RECALL 0.77, MCC 0.44; *curated jury score* ACC 0.66, PREC 0.66, RECALL 0.66, MCC 0.33)

**Supplementary figures labels**

**Supplementary Figure 1.** Distribution plots of 8 different input variable used by classifiers for the curated dataset. The values of the positive and negative sets are shown in blue and red respectively in panels A to G. Panels A, B, C, D, E, D and G show the distribution of Guildify scores, centrality values, betweenness values, function score, % of clusters K1, % of clusters LN, and clustering coefficient values respectively. Panel H presents the box-plots and a violin representation of the distribution of the shortest path values on the negative (orange) and positive (blue) sets.

**Supplementary Figure 2**. Box- and violin plots is AUC values on the three different classifiers. The different box-plots shown the distribution of the mean AUC values for the best models developed for each ADR using the three different classifiers: SVM (orange), Random Forest (Blue) and Neural Networks (green).

**Supplementary Figure 3.** B**ox- and violin plots for accuracy (ACC), precision (PREC), recall (REC), Receiver Operating Area Under Curve (ROC AUC) and MCC for the curated dataset.** Distribution of ACC, PREC, REC and ROC AUC values for individual classifiers: NN (green), RF (blue) and SVM (orange) as well as meta-predictions: consensus (cyan), jury-vote (magenta) and red-flag (red)

**Supplementary Figure 4. Heatmap of predictions at SOCs.** ACC, PREC, REC , ROC AUC and MCC values for predictions at SOCs for both individual classifiers (SVM, RF, NN) and voting (*jury vote*, consensus and *red flag f*or the controlled dataset.

**Supplementary Figure 5. Boxplots for Area Under Precision Recall Curve (AUPRC), Negative Predictive Value (NPV) and Positive Predicting Value (PPV) for DIAMOnD positive threshold**

**= 0.6 for the community dataset.**

Distribution of AUPRC, NPV, PPV values for individual classifiers: NN (green), RF (blue) and SVM (orange) as well as meta-predictions: consensus (cyan), jury-vote (magenta) and red-flag (red) for the different positive threshold and negative ratios (0.6-1.5, 0.6-3.0, 0.6-5.0)

**Supplementary Figure 6. Boxplots for Area Under Precision Recall Curve (AUPRC), Negative Predictive Value (NPV) and Positive Predicting Value (PPV) for DIAMOnD positive threshold**

**= 0.6 for the curated dataset.**

Distribution of AUPRC, NPV, PPV values for individual classifiers: NN (green), RF (blue) and SVM (orange) as well as meta-predictions: consensus (cyan), jury-vote (magenta) and red-flag (red) for the different positive threshold and negative ratios (0.6-1.5, 0.6-3.0, 0.6-5.0)

**Supplementary Figure 7. Boxplots for Area Under Precision Recall Curve (AUPRC), Negative Predictive Value (NPV) and Positive Predicting Value (PPV) for DIAMOnD positive threshold**

**= 0.7 for the community dataset.**

Distribution of AUPRC, NPV, PPV values for individual classifiers: NN (green), RF (blue) and SVM (orange) as well as meta-predictions: consensus (cyan), jury-vote (magenta) and red-flag (red) for the different positive threshold and negative ratios (0.7-1.5, 0.7-3.0, 0.7-5.0)

**Supplementary Figure 8. Boxplots for Area Under Precision Recall Curve (AUPRC), Negative Predictive Value (NPV) and Positive Predicting Value (PPV) for DIAMOnD positive threshold**

**= 0.7 for the curated dataset.**

Distribution of AUPRC, NPV, PPV values for individual classifiers: NN (green), RF (blue) and SVM (orange) as well as meta-predictions: consensus (cyan), jury-vote (magenta) and red-flag (red) for the different positive threshold and negative ratios (0.7-1.5, 0.7-3.0, 0.7-5.0)

**Supplementary Figure 9. Boxplots for Area Under Precision Recall Curve (AUPRC), Negative Predictive Value (NPV) and Positive Predicting Value (PPV) for DIAMOnD positive threshold = 0.8 for the community dataset.**

Distribution of AUPRC, NPV, PPV values for individual classifiers: NN (green), RF (blue) and SVM (orange) as well as meta-predictions: consensus (cyan), jury-vote (magenta) and red-flag (red) for the different positive threshold and negative ratios (0.8-1.5, 0.8-3.0, 0.8-5.0)

**Supplementary Figure 10. Boxplots for Area Under Precision Recall Curve (AUPRC), Negative Predictive Value (NPV) and Positive Predicting Value (PPV) for DIAMOnD positive threshold = 0.8 for the curated dataset.**

Distribution of AUPRC, NPV, PPV values for individual classifiers: NN (green), RF (blue) and SVM (orange) as well as meta-predictions: consensus (cyan), jury-vote (magenta) and red-flag (red) for the different positive threshold and negative ratios (0.8-1.5, 0.8-3.0, 0.8-5.0)

**Supplementary Figure 11. Boxplots for Area Under Precision Recall Curve (AUPRC), Negative Predictive Value (NPV) and Positive Predicting Value (PPV) for DIAMOnD positive threshold = 0.9 for the community dataset.**

Distribution of AUPRC, NPV, PPV values for individual classifiers: NN (green), RF (blue) and SVM (orange) as well as meta-predictions: consensus (cyan), jury-vote (magenta) and red-flag (red) for the different positive threshold and negative ratios (0.9-1.5, 0.9-3.0, 0.9-5.0)

**Supplementary Figure 12. Boxplots for Area Under Precision Recall Curve (AUPRC), Negative Predictive Value (NPV) and Positive Predicting Value (PPV) for DIAMOnD positive threshold = 0.9 for the curated dataset.**

Distribution of AUPRC, NPV, PPV values for individual classifiers: NN (green), RF (blue) and SVM (orange) as well as meta-predictions: consensus (cyan), jury-vote (magenta) and red-flag (red) for the different positive threshold and negative ratios (0.9-1.5, 0.9-3.0, 0.9-5.0)
